# Implementing a Novel Resident-Led Peer Support Program for Emergency Medicine Resident Physicians

**DOI:** 10.3390/bs15070943

**Published:** 2025-07-12

**Authors:** Kyra D. Reed, Alexandra E. Serpe, Alexandria P. Weston, Destiny D. Folk, Heather P. Kelker, Aloysius J. Humbert, Katie E. Pettit, Julie L. Welch

**Affiliations:** 1 Department of Emergency Medicine, Indiana University School of Medicine, Indianapolis, IN 46202, USA; dfolk@iu.edu (D.D.F.); hpetrase@iu.edu (H.P.K.); ahumbert@iu.edu (A.J.H.); kburdick@iu.edu (K.E.P.);; 2 Department of Emergency Medicine, Washington University in St. Louis School of Medicine, St. Louis, MO 63110, USA; serpe@wustl.edu; 3 Department of Emergency Medicine, Indiana University Health Arnett Hospital, Lafayette, IN 47905, USA; aweston1@iuhealth.org

**Keywords:** peer support, resident wellness, resident mental health, physician wellness, physician mental health, burnout, depression, emergency medicine, medical training, burnout

## Abstract

Background: Residency training is a formative and rigorous experience, with burnout rates reported at 76%. Formal peer support groups have shown improvement in burnout among healthcare workers with anxiety and depression. Objective: Implement a peer support program for emergency medicine (EM) residents and characterize utilization of metrics by demographics, burnout rates of participants, and overall session impact. Methods: An IRB-approved, longitudinal, prospective cohort study of 73 EM and EM/Pediatrics residents post-graduate year (PGY) 1–5 from July 2021–June 2022 was performed. Resident peer leaders were trained using a novel curriculum to lead peer support groups. Residents were invited to participate in biweekly sessions, with optional pre- and post-session surveys measuring demographics, burnout, themes discussed, and how they felt after sessions (Patients’ Global Impression of Change scale). Results: There were 134 attendances over 20 sessions, averaging 6 residents per session. Of 73 total residents, 37 (50%) participated at least once. All levels of training were represented, with half being female, 20% underrepresented in medicine, and 14% LGBTQ+. Overall burnout rates were unchanged for first-time attendances (49%, *n* = 18) vs. recurrent (50%, *n* = 11). Females had higher burnout at both baseline (60%, *n* = 15) and recurrent sessions (69%, *n* = 13). Following sessions, 94% of participants reported feeling immediately better and 100% of leaders felt prepared leading peer support sessions. Conclusions: This study demonstrates that residents utilize peer support, with many returning more than once. Despite stable burnout rates, 94% of participants felt immediately better after the session, suggesting that peer support is a valuable resource for residents actively experiencing burnout.

## 1. Introduction

Medical residency training is a demanding time for trainees and is associated with high rates of burnout (Eskander et al., 2021). Rates of burnout vary with one study reporting that 76% of EM residents reported burnout (Lin et al., 2019). Burnout is linked to depression and anxiety, increased medical errors, and decreased quality of patient care (Dewa et al., 2017; Eskander et al., 2021). A systematic review and meta-analysis revealed that 29% of residents experienced a major depressive episode during residency versus 8% of the general population (Folk et al., 2024). Physicians also have a significantly higher suicide rate than the general population (1.3% vs. 0.8%) (Folk et al., 2024).

To address these needs, the Accreditation Council for Graduate Medical Education (ACGME) has sought to provide resources to support resident well-being and identified resources that need to be developed to ensure an optimal learning environment (Daskivich et al., 2015). In describing the characteristics of the ideal learning environment to accommodate learners in times of stress, comments encompassed several themes: awareness and destigmatization of mental health issues, availability of mental health services, and a supportive culture (Daskivich et al., 2015).

The ACGME requires residency programs to take responsibility for optimizing resident well-being through wellness initiatives and accessible resources (ACGME, 2025). Barriers such as stigma and time constraints often limit residents’ ability to access wellness and mental health resources (Kolarik et al., 2018; Thomas et al., 2024). Although there are no standardized curriculums on how to optimize resident well-being, residency programs are starting to develop and research the utility of a well-being curriculum for trainees. Arnold et al. proposed a module-based curriculum for emergency medicine (EM) residents which incorporates a self-care series on sleep, nutrition, financial health, and mindfulness, as well as educating residents on physician suicide and how to access mental health care for themselves confidentially (Arnold et al., 2018). A multicenter prospective educational trial conducted at 10 EM residencies implemented a well-being curriculum and found no changes in burnout scores among resident physicians (Williamson et al., 2020).

One approach to improving well-being in medical residency training is by implementing a peer support program (Abrams, 2017). Peer support programs invite physician participants to listen, reflect, and reframe negative thoughts. They encourage group learning through wisdom sharing, coping, helpful resources, and a formal closing (Shapiro & Galowitz, 2016). Formal peer support groups have been effective for managing anxiety and depression (Broxterman et al., 2019). They have improved comradery among participants and reduced stigma around seeking mental health treatment (Miyamoto & Sono, 2012). Peer-led support groups have been utilized in different physician groups including COVID-19 pandemic support and litigation peer support for physicians involved in medical malpractice lawsuits (Doehring et al., 2023; Viswanathan et al., 2020). Peer support programs have had a positive impact on various physician groups which implies that it can positively influence mental health for resident physicians as well.

Small studies have emerged showing the utility of peer support for resident physicians. In 2021, one residency program created a near-peer support model for resident physicians to discuss mental health crises with a trained faculty member. Resident physicians felt that the peer support was useful and validating, and many resident physicians requested additional follow-up after the initial discussion with a faculty member (Ruzycki et al., 2024). A pilot study showed that group peer support helped to mitigate burnout symptoms among first-year internal medicine residents (Abrams & Granieri, 2018). Resident physicians who felt satisfied by peer support scored higher on residency milestone scores (Webber et al., 2021). Optimal peer support groups for resident physicians avoid fatalism and aim to foster intimate connections among residents (Jain et al., 2022). Moore et al. created a peer support program which offers peer support skills and trauma-informed care training to a diverse cohort of resident leaders, deployed these leaders to support their peers, and facilitated opportunities for participants to train other residents in these skills (Moore et al., 2024). Although small studies have demonstrated that peer support, and particularly near-peer models, may improve resident well-being, there is a paucity of research on sustainable, resident-led group models and their impact on EM resident well-being and burnout. Given the positive impact of peer support on physician mental health, we sought to create a standardized peer support program that is equitable and easily implementable for EM residents and characterize utilization metrics by demographics, burnout rates of participants, and overall session impact.

## 2. Methods

Study Design: We performed an institution-based IRB-approved, longitudinal, descriptive, prospective cohort survey study of 73 EM and EM/Pediatrics residents PGY 1–5, between June 2021–June 2022.

Study Setting and Population: This study was set within a large emergency medicine (EM) residency, consisting of 73 total residents, with 63 being categorical EM, and 10 EM/Pediatrics (EM/Peds) residents. The primary training sites involved three Level 1 Trauma centers including county, academic, and pediatric hospitals.

Study Protocol: Peer support participants, including EM and EM/Peds residents, were recruited via email introducing the program, which provided information about the peer support program and details of the study. Participation was voluntary and consent was obtained for all leaders and participants. Rolling enrollment was utilized to be as inclusive as possible and accommodate varying resident schedules and needs. Peer support sessions were scheduled biweekly immediately following residency didactics. Each in-person session was held in a private space with the option for participants to join virtually to ensure accessibility and flexibility.

**Figure 1:**
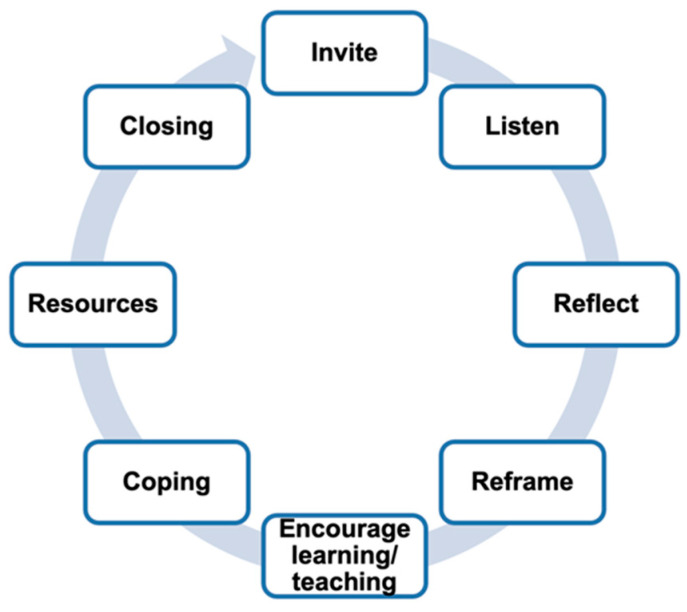
Fundamentals of peer support.

Both the peer support session structure and peer leader training followed the model of the National Alliance on Mental Illness (NAMI) peer support program, which has been adapted for physicians and successfully piloted in EM settings (Connors et al., 2023; Doehring et al., 2023). The one-hour peer support sessions follow an intentional structure (Figure 1) guided by a peer support group leader and include key principles such as inviting participants to listen, validating emotions, empathizing, reflecting, and reframing perspectives (Shapiro & Galowitz, 2016). Additional components of sessions include learning through shared group wisdom, encouraging positive coping strategies, providing helpful resources, and ending with a formal closing. Thus, session flow followed the general format of the following: (1) check in from all participants in the session, (2) open discussion facilitated by the leaders based on identified themes from the check in, and (3) check out where participants could reflect on themes that resonated or helpful strategies to implement before the next session. Rules and guidelines for peer support were reviewed with participants before starting the sessions, which included reminding participants of confidentiality, that peer support is not formal therapy, and the session will not go beyond one hour in duration.

The biweekly intervention allowed for two peer support group sessions per residency block. The content for the peer support groups was determined by the resident leader, who used the “check-in” portion of the session to identify themes of experiences and stressors shared by the group. The leader would then invite the group to expand on their feelings, perspectives, and coping strategies for these themes. Importantly, the peer leader encourages participants to share group wisdom, especially for varying levels of training to reflect on strategies that helped with different scenarios. If there was any difficulty in identifying themes after the check-in portion, leaders were encouraged to use a set of topics to fuel discussion, including common issues in residency training such as transitions between levels of training and responsibilities, work–life balance, time management strategies, team communication, leadership challenges, etc. For closing the session, leaders invited everyone to share something positive, usually either something the participant is looking forward to, or a moment from the session that was impactful. Mental health and wellness resources were then offered. Leaders kept the session to one hour to respect each participant’s time.

Peer leaders were trained using a combination of (1) asynchronous material review and (2) a one-hour virtual group meeting. Peer leader asynchronous training included an informational video and literature on burnout and peer support. The group training session was led by a peer support faculty mentor, utilizing a facilitator curriculum that was designed and implemented from resources provided by the National Alliance on Mental Illness and the guidelines for peer facilitators from the American Medical Association (Connors et al., 2023; Jin & Chaudhari, n.d.). This leader training program model was successfully developed in previous emergency medicine peer support group programs to train faculty leaders (Connors et al., 2023; Doehring et al., 2023). The training session covered the basics of how sessions are structured, the standard rules and regulations of sessions, participant safety planning for mental health emergencies, administering pre and post surveys, and discussing the skills of listening, validating, and reframing. For safety planning, leaders were informed of the process to activate a designated faculty member that was on call as a resource for peer support leaders for any acute issues or safety concerns that arose during the session. This was followed by an open forum for questions.

Key Outcome Measures: Participants were asked to complete anonymous, voluntary, electronic pre-session and post-session surveys that included validated and customized questions. The pre-session survey was either a baseline survey before the first peer support session or a recurrent survey that included demographic items and a burnout measure. Pre-session surveys were dichotomized as either “Baseline” or “Recurrent” sessions for the participants in order to track differences in aggregate mental health metrics for those returning to peer support sessions. A baseline session refers to the first time a participant has been to a session, and the survey was given before the start of the first session that was attended. Recurrent session refers to anything other than the first session attendance for that participant, and was given before the start of the session. We used the validated non-proprietary Maslach Burnout Inventory (MBI) Equivalent Single-Item Scale (Dolan et al., 2015) that instructed respondents to rate their level of burnout based on their own definition, with responses scored on a five-category scale (Table 1). For our study, this burnout scale was dichotomized as a score ≤ 2 (no symptoms of burnout) versus a score ≥ 3 (positive symptoms of burnout). The MBI equivalent single-item scale is easy to interpret, with the response scale explicitly indicating where a change in values signals symptoms of burnout (3 = “I am definitely burning out”) versus no burnout (2 = “don’t feel burned out”) (Dolan et al., 2015).

The post-session survey included the Participant-Rated Global Impression of Change (PGIC) (Kroenke et al., 2018), which is a single question item that asks, “Since the start of the session, overall I am feeling…”, with answers on a 7-point Likert scale of much better to much worse. The PGIC is a participant-reported outcome that assesses perceived change over time in symptoms. The PGIC scale shows moderate test–retest reliability and demonstrates responsiveness to clinical change (Eremenco et al., 2022). It is a retrospective scale that does not indicate changes in specific domains. Post-session surveys for leaders included reporting the number of participants for each session, which provided the total attendance number.

Data Analysis: Descriptive statistics were used to characterize peer support utilization by demographics, rates of burnout, and the PGIC measure. Survey data was collected via Qualtrics and statistical analysis was performed using SAS version 9.4 (SAS Institute, Cary, NC, USA). Demographic domains were calculated as a percentage to assess whether groups were representative of the residency as a whole. Burnout measures were calculated as a percentage, with a binary cutoff point of ≥3 considered positive. Chi-Squared and Fishers exact tests were used to examine differences between groups. For PGIC, responses that scored ≥ 5 (i.e., 5 = slightly better, 6 = moderately better, and 7 = much better) were included in the calculation for feeling better after the session.

## 3. Results

From July 2021 through June 2022, there were 20 resident-led peer support group sessions held following resident didactics. These sessions were biweekly and conducted virtually, in-person, or hybrid depending on the group’s needs. From the peer leader post-session surveys for each session (*n* = 20), there were 134 total resident attendances, with an average of 6 residents per session. Of those attendances, 59 pre-session surveys were completed, comprising both Baseline/First-Time Attendance (*n* = 37), and Recurrent Session Surveys (*n* = 22). Given the baseline survey data, of the 73 EM and EM/Peds residents total, a minimum of 50% participated at least once. All levels of training were represented in baseline survey data, and 41% self-identified as female, 20% Underrepresented in Medicine (URM), and 14% LGBTQ+. The participant sample in the study was shown to be closely representative of the residency program (Table 2). Recurrent survey data is an aggregate of all participants.

Of those respondents answering the question on burnout, which was less than those answering demographics questions, overall burnout rates were unchanged for those attending peer support for the first time (49%, n = 18) and for those completing recurrent peer support surveys (50%, n = 11) (Table 3). Females had higher burnout at baseline (60%, n = 15) and significantly higher burnout in recurrent session surveys (69%, n = 13).

Additionally, 47 post-session surveys were completed, with 94% of participants reporting feeling better immediately after the session (Table 4). Changes over time for PGIC or Non-proprietary Maslach Burnout Inventory (MBI) Equivalent Single-Item Scale were unable to be calculated or remarked upon because specific individuals were not followed over time.

The open-ended question and qualitative feedback provided helpful quotes regarding peer support. One resident stated, “This was the best use of wellness time we have ever had.” Another remarked, “It was nice to hear others going through the same fears and struggles.” One resident posted on a public forum that “Yesterday in peer support counseling I remarked, “the last thing dozens of people with COVID saw was me, upside down, in goggles, two masks and a gown as I prepared to intubate them,” and wow. I never paused to reflect on how profoundly sad that is. So very thankful that my program leaders strong arm us into talking about and processing the things we see and treat!”

## 4. Discussion

This intervention was easily implemented with minimal resources and showed significant engagement with 134 attendances over a one-year period. Given that 94% of participants reported feeling better immediately after the session, this suggests that a peer support group intervention has a positive impact for participants after a single, one-hour session. Additionally, 100% of resident peer leaders that were still in training returned as leaders the following year and the total number of leaders doubled, further adding to the feasibility of program implementation and perceived utility by residents.

Trends in participant demographics, including returning to subsequent sessions for individuals identifying as females or LGBTQ+, were noted. However, for individuals identifying as males, interns, or URM, there appeared to be a decrease in return rate for subsequent sessions. This is difficult to interpret, however, given the recurrent session surveys are in aggregate and did not follow a specific individual over time. Regardless, to address this drop in participation for interns, next steps will be to expand the program to include Intensive Care Unit (ICU) peer support group sessions for the EM interns, including protected time from clinical duties to attend with food provided. This will ideally address the barriers in attending sessions during intern year by bringing peer support to the ICU rotation. Other areas to address would be retaining URM and male residents. We intend to champion this intervention in a variety of settings, including grand rounds and orientation to facilitate accessibility and exposure to the program. Additionally, more investigation into strategies for tailoring wellness needs to each of these groups is needed. Focus groups would lend information in this area.

Our initial hypothesis was that attendance at peer support would decrease burnout, which was not seen over the first year of implementation in our study. However, despite no change in burnout from baseline in pre-session surveys, 94% of participants reported feeling better immediately following the sessions, suggesting that peer support can be used as a resource for those actively experiencing burnout. Burnout remained stable for those that participated in multiple sessions, possibly suggesting that those individuals with burnout continued to seek peer support as a resource. Our study’s results showing no changes in burnout for most participants contrasts with a prior pilot study on internal medicine residents which showed that peer support did improve burnout symptoms among interns. Interestingly, females had higher rates of burnout after recurrent peer support sessions in our study. It is known that females in EM continue to report higher emotional exhaustion and a lower sense of personal accomplishment than their male counterparts (Thakur et al., 2025). A prior study (Williamson et al., 2020) showed no changes to resident well-being after implementation of a curriculum for EM residents, but no prior studies to our knowledge have shown that peer support increases burnout among females. In fact, existing literature supports that the implementation of a physician-focused peer support program improves physician well-being and departmental culture (Tolins et al., 2023). Therefore, the impact of peer support on burnout longitudinally warrants further study through a randomized controlled trial with a larger sample size.

The rolling enrollment strategy allowed for the immediate goal of removing barriers to mental health support by providing every resident with the opportunity to experience the potential benefit of the sessions. Understanding the chaotic and unpredictable nature of resident schedules, this enrollment style allowed for flexibility in participation in the study.

Our approach targeted all learners regardless of social identity in the initial implementation. We know that those who identify with minoritized identities experience unique stressors and utilize different coping strategies compared to non-minoritized groups (Drabble et al., 2018). Thus, it follows that a thoughtful approach to wellness and mental health with a prospective lens on diversity, equity, and inclusion efforts would be a reasonable next step in expanding the peer support program. This could include subgroups of peer support based on interest and need, along with additional training for leaders to help navigate challenging scenarios that may arise during peer support.

## 5. Limitations

There are several key limitations to this study that could have introduced bias, potentially limiting the generalizability of this study. Survey studies inherently have response and selection bias and can be influenced by survey fatigue. In this study, the demographics of the study group in baseline surveys reflected the demographics of the residency program. Individual participants were unable to be tracked over time, given the respondents used different self-made identifiers or none at all. This impacted the conclusions that could be made in the recurrent session surveys.

The number of participants completing surveys in this study was low compared to the known number of attendances. The rolling enrollment nature of the study and reliance on self-initiated survey completion were known limitations. Additionally, when it was discovered that access to the surveys was challenging in the first few sessions, a QR code was created for in-person sessions, while links to surveys were added to chats during virtual meetings to enhance response rates. Navigating the distribution of survey links and QR codes at the beginning and end of the sessions will be incorporated into the next peer leader orientation. Further, not all questions were mandatory to answer for the surveys, leading to variability in response rate.

Developing this program required minimal time and effort up front. However, when scheduling the sessions, it became clear no ideal time for peer support sessions exists given the complexity of resident schedules. On a mid-year poll of residents, it was determined that residents preferred to keep the sessions after didactics to encourage the most participation, despite understanding some residents will have to leave for emergency department shifts, which was a limiting factor. Trialing sessions during didactic time did increase participation and was well-received. However, given every residency training program has a different scheduling structure, finding the best time and platform (in-person, virtual, or hybrid) to hold the sessions will be program specific. Also, this peer support program was performed in two residency programs at a single institution, which may limit generalizability at this time.

Lastly, stigma remains a barrier for residents seeking mental health resources in general. This could be an inherent limitation in openness to participation that will be attempted to be addressed with further mental health education, standardization of resources, proactive strategies, implementation of peer support into didactics, and general culture change regarding physician mental health moving forward.

## 6. Conclusions

A resident-led peer support program was successfully implemented as a resource for EM and EM/Peds residents actively experiencing burnout and can serve as a framework for other residency programs. In the future, we plan to develop additional approaches to peer support, including tailoring to specific learner groups, expanding to additional residency programs, and analyzing individual participant data over time to assess long-term benefits on burnout and other mental health metrics. Additionally, we plan to assess the impact on the peer support leaders and continue to improve accessibility to leader training and peer support sessions.

## Figures and Tables

**Table 1:** Non-proprietary Maslach Burnout Inventory (MBI) Equivalent Single-Item Scale.

“Overall, based on your definition of burnout, how would you rate your level of burnout?”	
1	“I enjoy my work. I have no symptoms of burnout”
2	“Occasionally I am under stress, and I don’t always have as much energy as I once did, but I don’t feel burned out”
3	“I am definitely burning out and have one or more symptoms of burnout, such as physical and emotional exhaustion”
4	“The symptoms of burnout that I’m experiencing won’t go away. I think about frustration at work a lot”
5	“I feel completely burned out and often wonder if I can go on. I am at the point where I may need some changes or may need to seek some sort of help.”

**Table 2:** Demographics of resident peer support group participants (*n* = 59) for first-time and recurrent sessions.

Demographics	Overall Residency Program Demographics (n = 73)	First-Time (n = 37)	Recurrent (n = 22)
Male	39 (53%)	22 (59%)	9 (41%)
Female	34 (47%)	15 (41%)	13 (59%)
URM ^a^	9 (12%)	8 (20%)	3 (13%)
LGBTQ+	Unknown	5 (12%)	5 (22%)
PGY 1 (Interns)	23 (32%)	12 (29%)	3 (13%)
PGY2–5 (Upper Levels)	50 (68%)	24 (58%)	19 (82%)

^a^ URM = Black/African American, Hispanic/Latino, Native Hawaiian or other Pacific Islander, American Indian/Alaska Native.

**Table 3:** Reported burnout of resident peer support group participants for first-time and recurrent sessions based on demographics ^a,b^.

			No Burnout (MBI Score =< 2)	Burnout (MBI Score >= 3)	*p*-Value
First-Time (N = 36)					
	Gender				0.1787
		Female	6 (40.0)	9 (60.0)	
		Male	13 (68.42)	7 (41.18)	
		Prefer not to say	0 (0.00)	1 (100.00)	
	Race				0.5858
		Non-white	7 (63.64)	4 (36.36)	
		White	12 (52.17)	11 (47.83)	
		Prefer not to say	0 (0.0)	1 (100.0)	
	LGBTQ				0.1914
		No	15 (51.72)	14 (48.28)	
		Yes	4 (80.0)	1 (20.0)	
		Prefer not to say	0 (0.0)	2 (100.0)	
	Total		19 (52.78)	17 (47.22)	0.6317 ^c^
Recurrent (N = 20)					
	Gender				0.0198 *
		Female	3 (25.0)	9 (75.0)	
		Male	7 (87.50)	1 (12.50)	
	Race				1.00
		Non-white	1 (50.0)	1 (50.0)	
		White	9 (50.0)	9 (50.0)	
	LGBTQ				0.3034
		No	9 (60.0)	6 (40.0)	
		Yes	1 (20.0)	4 (80.0)	
	Total		10 (50)	10 (50)	0.6317 ^c^

^a^ Burnout measure using the non-proprietary MBI single-item burnout measure. ^b^ Percent burnout calculated by number of participants by gender identity reporting burnout by total participants by gender identity responding to each survey. The *p* values are derived from paired t-tests. * *p* < 0.05 indicates significance. ^c^ Compares the total first time and recurrent.

**Table 4:** Post-session Patients’ Global Impression of Change (PGIC) scale.

Question	N	Much Better	Moderately Better	A Little Better	No Change	A Little Worse	Moderately Worse	Much Worse
Since the start of the session, overall I am feeling…	47	14 (29.8%)	17 (36.2%)	13 (27.7%)	2 (4.3%)	1 (2.1%)	0 (0.0%)	0 (0.0%)

## Data Availability

The datasets presented in this article are not readily available due to participant privacy.

## Abbreviations

The following abbreviations are used in this manuscript:

EM	Emergency Medicine
URM	Underrepresented in Medicine
PGIC	Participant-Rated Global Impression of Change
NAMI	National Alliance on Mental Illness
